# Editorial: Achieving health equity: sustainability of plant-based diets for human and planetary health

**DOI:** 10.3389/fpubh.2023.1285161

**Published:** 2023-09-28

**Authors:** Christopher D. Gardner, Peggy Policastro, May C. Wang

**Affiliations:** ^1^Department of Medicine, School of Medicine, Stanford Prevention Research Center, Stanford University, Stanford, CA, United States; ^2^New Jersey Institute for Food, Nutrition, and Health, Rutgers School of Environmental and Biological Sciences, The State University of New Jersey, New Brunswick, NJ, United States; ^3^Fielding School of Public Health, University of California, Los Angeles, Los Angeles, CA, United States

**Keywords:** plant-based diet, planetary health, human health, equity, climate change

One of the most urgent public health issues facing the human race is climate change due to global warming. Climate change affects health in many ways including heat-related illnesses, infectious diseases, and lack of access to clean water. While it affects everyone, the poor and marginalized will disproportionately suffer the effects, further widening health inequities.

*What has led to global warming?* The Intergovernmental Panel on Climate Change, made up of scientific experts from all over the world, concluded that human activities have contributed to the increase in greenhouse gas (GHG) emissions that has led to global warming. About one-third of all GHS emissions are food-related, with a high proportion of these emissions coming from agriculture and land use (e.g., methane from the digestive process of livestock, nitrous oxide from fertilizers, carbon dioxide from deforestation). Food storage and processing, food transport and the management of food waste contribute to a much smaller proportion of GHG emissions (1). In an impressive analysis of data from more than 38,000 farms in over 100 countries, Poore and Nemecek (2) concluded that there are huge differences in GHG emissions of different foods with beef being the highest emitter of GHG, due mostly to land use and farm-stage processes (not transport). In general, animal-based food emits more GHG than plant-based food. Hence, from an environmental perspective, a shift from animal-based to plant-based diets has the potential to contribute significantly to ameliorating the effects of climate change. From a human health perspective, such a shift would align with current dietary guidelines which recommend increased intake of fruits and vegetables, whole grains, legumes and nuts and decreased intake of red meat, sugar and refined grants. Such a recommendation was described by the EAT-Lancet Commission of Food, Planet and Health which produced in 2019 the first full scientific review of what constitutes a healthy and sustainable diet (3). This recommendation which implies a transformation in the food system has led to increased interest in the development of new plant-based food products. Indeed, the food industry has been developing and marketing new plant-based food products (e.g., non-dairy milks made from nuts and legumes, and meatless beef substitutes), as consumer demand grows for plant-based foods, driven to a considerable extent by Gen Z and Millennials. According to the Plant-based Foods Association, sales of plant-based foods and beverages reached US$7 billion for the year 2021, a 27% increase from 2019.

*What are the human and planetary health benefits of increasing the production of plant-based foods and diets?* Research on the long-term health and environmental impacts of shifting from animal-based diets to plant-based diets is just emerging, and there is currently a lack of evidence and a clear framework for guiding translational research on plant-based diets.

This Research Topic is a collection of papers on the human health and planetary (environmental) health aspects of plant-based foods and diets including the nutrient composition of plant-based beverage products. The mini review by Wong et al. highlights the potential role of plant-based foods in reducing risk of chronic illness and disability due to infections caused by viruses such as SARS -COV-2 (COVID-19), Human Papillomavirus Virus (HPV), and Hepatitis C Virus (HCV). Another mini review by Ahmad discusses the existing literature on the role of plant-based diets in treating obesity. Both of these articles conclude that while there is some evidence to support the health benefits of plant-based foods in reducing risk of communicable and non-communicable diseases, studies using more robust study designs including longer follow-up duration, are needed. The articles by Walther et al. and Smith et al. conclude that the nutrient composition of plant-based milks (beverages) is not comparable to that of cow's milk. Studies of their impact on human nutrition especially of vulnerable populations such as growing children are lacking. Finally, Espinosa-Marron et al. use a socio-ecological framework to discuss how national, state and municipal policies affect food systems and sustainability. The article addresses the need for multi-sector collaborations as policy solutions are sought to address food-related climate change concerns so as to not overlook unintended consequences that may arrest efforts to achieve health equity.

For decades, public health researchers have recognized the need for closer collaborations across sectors. Such collaborations are necessary for disciplines to more effectively address the impact of food on health through agricultural, economic and health policies and programs that are better aligned to achieve health equity. The consequences of human activities on human and planetary health can no longer be ignored, and there is an urgent need to heed the recommendations of the EAT-Lancet Commission and the Intergovernmental Panel on Climate Change. Implementation of these recommendations will require that we rethink how we conduct research and train the next generation of scientists to become effective at integrating knowledge and skills across sectors and disciplines.

## Author contributions

CG: Writing—review and editing. PP: Writing—review and editing. MW: Writing—original draft.
